# Transfer of monolayer TMD WS_2_ and Raman study of substrate effects

**DOI:** 10.1038/srep43037

**Published:** 2017-02-21

**Authors:** Jerome T. Mlack, Paul Masih Das, Gopinath Danda, Yung-Chien Chou, Carl H. Naylor, Zhong Lin, Néstor Perea López, Tianyi Zhang, Mauricio Terrones, A. T. Charlie Johnson, Marija Drndić

**Affiliations:** 1 Department of Physics and Astronomy, University of Pennsylvania, Philadelphia, Pennsylvania 19104, USA; 2 Department of Electrical and Systems Engineering, University of Pennsylvania, Philadelphia, Pennsylvania 19104, USA; 3 Department of Materials Science and Engineering, University of Pennsylvania, Philadelphia, Pennsylvania 19104, USA; 4 Department of Physics, The Pennsylvania State University, University Park, Pennsylvania 16802, United States; 5 Center for 2-Dimensional and Layered Materials, The Pennsylvania State University, University Park, Pennsylvania 16802, United States; 6 Department of Materials Science and Engineering, The Pennsylvania State University, University Park, Pennsylvania 16802, United States; 7 Department of Chemistry, The Pennsylvania State University, University Park, Pennsylvania 16802, United States

## Abstract

A facile transfer process for transition metal dichalcogenide WS_2_ flakes is reported and the effect of the underlying substrate on the flake properties is investigated using Raman spectroscopy. The flakes are transferred from their growth substrate using polymethyl methacrylate (PMMA) and a wet etch to allow the user to transfer the flakes to a final substrate using a microscope and micromanipulator combined with semi-transparent Kapton tape. The substrates used range from insulators such as industry standard high-k dielectric HfO_2_ and “green polymer” parylene-C, to conducting chemical vapor deposition (CVD) grown graphene. Raman spectroscopy is used first to confirm the material quality of the transferred flakes to the substrates and subsequently to analyze and separate the effects arising from material transfer from those arising from interactions with the substrate. We observe changes in the Raman spectra associated with the interactions between the substrates in the flakes. These interactions affect both in-plane and out-of-plane modes in different ways depending on their sources, for example strain or surface charge. These changes vary with final substrate, with the strongest effects being observed for WS_2_ transferred onto graphene and HfO_2_, demonstrating the importance of understanding substrate interaction for fabrication of future devices.

Two dimensional transition metal dichalcogenide (TMD) materials are an important addition to the two-dimensional material family as they fill in the gap between conducting graphene and insulating boron nitride. Monolayer tungsten disulfide (WS_2_) in particular, with a direct band gap of 2.1 eV^1^, is interesting due to its predicted strong spin orbit coupling in the single layer^2^,^3^ and potential for use in optoelectronics with a measured photoluminescence that is stronger than MoS_2_^4^,^5^.

Each of these applications will require the use of different substrates that have the potential to enhance or degrade the native properties of the WS_2_ flakes. It has been shown in graphene^3^,^6^,^7^,^8^ and MoS_2_^8^,^9^,^10^,^11^,^12^,^13^,^14^ that the device substrate and capping oxides can have an effect on the material properties, including strengthening the spin-orbit interaction^3^, enhancing transport properties^11^, and tuning the band gap^7^,^9^,^14^. One powerful method for probing changes in material properties is by analyzing shifts in the phonon peaks of the material’s Raman spectrum^5^,^6^,^7^,^9^,^10^,^12^,^13^,^15^,^16^,^17^,^18^,^19^,^20^,^21^,^22^,^23^,^24^,^25^,^26^,^27^,^28^. The shifts in the Raman spectrum come from sources such as interface effects which create stress and strain^7^,^8^,^10^,^13^,^15^,^16^,^17^,^18^,^21^,^22^,^23^,^24^,^25^,^26^,^29^ or charge doping^5^,^19^,^20^, and defects^27^,^28^. In the case of WS_2_ layered on MoS_2_^13^,^21^, for example, interlayer coupling can occur and is observable as shifts and enhancements in the out-of-plane Raman modes and in the photoluminescence. The interlayer coupling also alters the band alignment between the two materials, further tuning the optical and electrical properties of the MoS_2_-WS_2_ heterojunction^30^.

In previous studies, Raman spectra have been measured of WS_2_ on substrates such as SiO_2_^5^,^15^,^16^,^20^,^24^,^31^,^32^,^33^,^34^,^35^,^36^,^37^,^38^,^39^,^40^,^41^,^42^,^43^,polyethylene terephthalate^24^, Al_2_O_3_^19^, sapphire^15^,^39^, boron nitride^18^,^25^,^44^, graphene^18^, MoS_2_^13^,^21^, and quartz^41^. However, the previous studies primarily used the Raman spectra just to confirm the presence of hexagonal WS_2_ by roughly locating the peaks at the expected positions, but did not look closely for any variations in the peaks owed to substrate effects or changes in the material quality. One exception, L. Su *et al*.^15^, looked at two types of substrates, SiO_2_ and sapphire, however it focused largely on the differences in Raman shifts between as-grown structures. Currently, no experimental study of variations in the Raman spectra of WS_2_ from transfer to a large number of substrate types has been reported, to our knowledge. In MoS_2_ and WSe_2_^14^, the choice of substrate has in fact been shown to have a significant impact on the Raman and thus properties of the material, further motivating our study of these effects on WS_2_.

In this study, facilitated by an easy and fast transfer method, we present a focused study on the effects of transfer and substrates on the resulting material properties as determined with Raman spectroscopy. We demonstrate a flake transfer and positioning method based on previously published methods^32^,^37^,^44^,^45^,^46^,^47^,^48^,^49^,^50^. Our method is most similar to that used for graphene by Zomer *et al*.^48^, but uses a polymethyl methacrylate (PMMA) capping layer and potassium hydroxide (KOH) etch to remove the WS_2_ from the growth substrate and Kapton tape, which leaves minimal residue and loses its adhesion in acetone, as the tape sticking the PMMA to a glass slide. The Kapton tape also serves a dual purpose in helping to create a transfer assembly which secures the location and surface contact of the PMMA/flakes to the final substrate, resulting in a transfer success rate above 84 percent for flakes transferred for this study (44 of 52 flakes), and with a single flake positioning accuracy of <10 microns, without needing a heated microscope stage^47^,^48^,^49^. To understand the effect of substrate we use a variety substrates of interest for nanofabrication, specifically SiO_2_, glass slides, HfO_2_, graphene, and parylene-C. By comparing the location, full width at half maximum (FWHM), and amplitudes of both in-plane and out-of-plane Raman modes, before and after transfer and after a subsequent annealing step, we are able to analyze and empirically separate the effects arising from material transfer from those arising from interactions with the substrate on the phonon characteristics of monolayer WS_2_.

## Results

**Figure 1:**
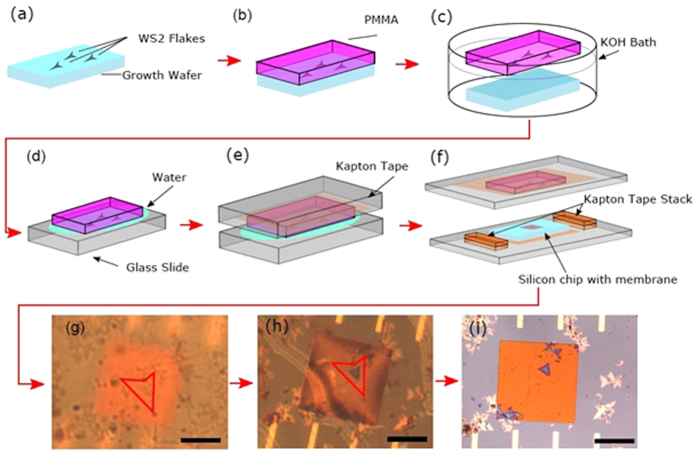
Diagram and optical images of a flakes transfer process. (**a**) WS_2_ flakes are grown on a SiO_2_ surface. (**b**) PMMA is spun onto the WS_2_ growth substrate. (**c**) The substrate with PMMA resist is placed into 1 M KOH heated to 70 °C which dissolves the SiO_2_ allowing the PMMA/flakes to be released to float on the surface of the KOH. (**d**) Using a glass slide the floating PMMA is removed from the KOH and transferred to a water bath for 30 minutes and then again, using a glass slide, is removed from the water and allowed to float on top of the glass slide. (**e**) A glass slide with double-sided Kapton tape is placed and pressed over the floating PMMA to secure it to the tape. (**f**) The glass slide with the PMMA is aligned over a target substrate secured to a separate glass slide and has two Kapton tape pillars that attach the two slides together upon contact. (**g**) The desired flake during alignment is visible under the optical microscope through the glass, tape, and PMMA and is outlined in red. A micromanipulator is used to move the flake to the desired position, with a Si_3_N_4_ window in the background. (**h**) The flake has been lowered onto the sample and pressure has been applied to secure the two slides together, providing good contact between the flake and the final substrate surface, as evidenced by the substrate surface and flake being in the same focal plane. (**i**) Si_3_N_4_ window and placed flake post overnight Acetone bath. All scale bars are 40 microns.

The transfer process, shown in Fig. 1, combines aspects of previous methods, used for both WS_2_ and other two dimensional materials, with some new aspects. The previously reported aspects are specifically the etching of SiO_2_^32^,^37^,^45^,^46^ to remove the flakes from the growth substrate and the use of micromanipulation of flakes on PMMA under a microscope^44^,^47^,^48^,^49^,^50^. The flakes are CVD grown on a SiO_2_/Si graphics, Fig. 1(a), as described in the Methods section. They are isolated from the growth substrate by spin coating PMMA, Fig. 1(b), and etching away the silicon oxide with 1 M KOH over a period of 2 hours, Fig. 1(c). The etching is followed by washing the PMMA/flakes of residual KOH in an H_2_O bath, and finally they are lifted out of H_2_O to float on water on a glass slide (Fig. 1(d)), flakes facing down. Where the process begins to differ from previous methods is the use of double sided Kapton tape on a separate glass slide to pick up the PMMA/flakes from the glass slide, Fig. 1(e).

The final substrate for transfer is attached to a separate glass slide with double-sided Kapton tape, and two stacks of double-sided Kapton tape with the same total thickness as the substrate are placed on either side of it as depicted in the bottom of Fig. 1(f). This assembly is placed on and centered under a microscope. Next, the PMMA/flake slide is attached, facing downwards, to a plastic arm which is then installed on a micro-manipulator stage and positioned over the substrate such that the desired flake is above the desired final position, as depicted in Fig. 1(f) and shown in the supplementary information Fig. S1.1(a–c). The microscope stage is then carefully raised. When the substrate, in this case a Si_3_N_4_ window, comes into focus, Fig. 1(g), adjustments to its position can be made. If the positioning is satisfactory, the substrate is raised until the surface comes into contact and is in focus with the desired flake, Fig. 1(h). The tape stacks on the sides of the chip as well as any exposed Kapton tape on the PMMA slide attach both slides together and the whole assembly can be removed from the microscope setup, supplementary information Fig. S1.1(d,e). The assembly is placed on a hot plate set to 175 °C for 20 minutes to further ensure contact, supplementary information Fig. S1.1(f). It is then allowed to cool down in air for 5 minutes and placed in an acetone bath for at least 12 hours to dissolve the PMMA, supplementary information Fig. S1.1(g). After the PMMA has been dissolved, the sample is removed, cleaned with fresh acetone and isopropanol, and dried with N_2_ gas, Fig. 1(i). In order to further study the surface interaction, the sample is finally annealed under Ar:H_2_ (5:1) gas mixture at 300 °C for two hours. This annealing step has been shown to be crucial in removing excess PMMA and promote surface adhesion^9^,^21^,^51^. Specifically in the case of MoS_2_^51^, annealing times in excess of 90 minutes are shown, via Raman and PL, to have increased the post transfer substrate bonding, creating an increased strain on the monolayer TMD.

**Figure 2:**
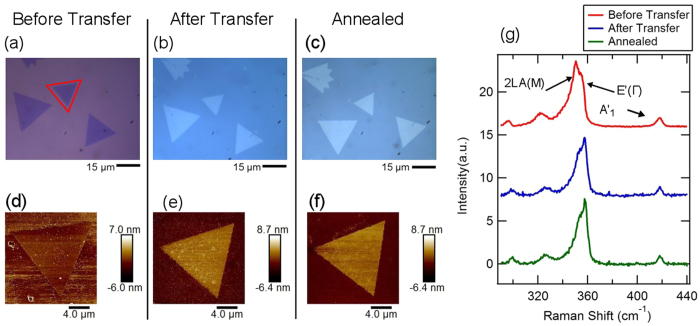
Optical and AFM images, with associated Raman spectra, for a monolayer WS_2_ flake before and after transfer. (**a**) Optical image of flake, pre-transfer, on growth substrate. (**b**) AFM image of flake in (**a**) before transfer. (**c**) Raman spectrum of flake in (**a**) showing the characteristic WS_2_ peaks at 351, 356, and 417 cm^−1^. (**d**) Optical image of flake post transfer, on the silicon oxide substrate. (**e**) AFM of flake in (**d**). (**f**) Raman spectrum of the transferred flake in (**d**) showing the same characteristics peaks as in (**c**).

It is important to show that the transfer method does not dramatically degrade the material. We therefore performed a detailed analysis of a monolayer flake, Fig. 2, as it went through the transfer process. The flake was transferred from the original 300 nm SiO_2_/Si wafer growth substrate to a 150 nm SiO_2_/Si wafer substrate. In order to check the quality of the transferred WS_2_, we characterized the flake optically Fig. 2(a–c), with atomic force microscopy Fig. 2(d–f), and using Raman spectroscopy Fig. 2(g) before transfer, after transfer, and post thermal annealing. Figure 2(a–c), with a monolayer flake (bordered in red), shows that optically, there is no change in the flake throughout the transfer process. Inspection of the flakes by AFM shown in Fig. 2(d–f), indicates that some creases have formed in the flake post transfer and that the edges have been damaged by the KOH etching, but the flake is otherwise intact. The importance of the annealing step is shown in Fig. 2(f) compared to Fig. 2(e), where in Fig. 2(f) there is a marked decrease in the leftover PMMA on the surface. We therefore conclude the transfer process causes minimal damage to the flakes and that the thermal annealing is a necessary step for improving transfer quality.

The Raman data of the monolayer before transfer, shown in red as the top trace of Fig. 2(g), taken using an excitation of 532 nm with a spectrometer resolution of 0.5 cm^−1^, shows the typical spectrum for monolayer WS_2_ in range of 290 to 440 cm^−1^ (14,35)^15^,^36^. The most prominent modes that are observed in this range are the in-plane acoustic 2LA(M) at 350 cm^−1^, in-plane optical E’(Γ) at 356 cm^−1^, and out-of-plane optical A’_1_ at 418 cm^−1^. The absence of the multilayer 311 cm^−1^ Raman peak further verifies that the flake is monolayer^41^. The after transfer (blue) and post annealing (green) Raman spectra show a blue shift in the in-plane peaks (2LA(M) and E’(Γ)) as well as a change in the relative ratio of peak heights with the E’(Γ) peak at 356 cm^−1^ increasing in amplitude compared to the others.

In order to investigate these changes more in depth, we have measured the Raman spectra of monolayer WS_2_ transferred from its growth substrate to a range of final substrate types including 300 nm SiO_2_ on silicon, 20 nm HfO_2_ on 300 nm SiO_2_/Si, 50 nm parylene-C on 300 nm SiO_2_/Si, optically transparent glass, and graphene. For each substrate we measure the Raman spectra of several monolayer flakes before transfer, after transfer, and post annealing.

**Figure 3:**
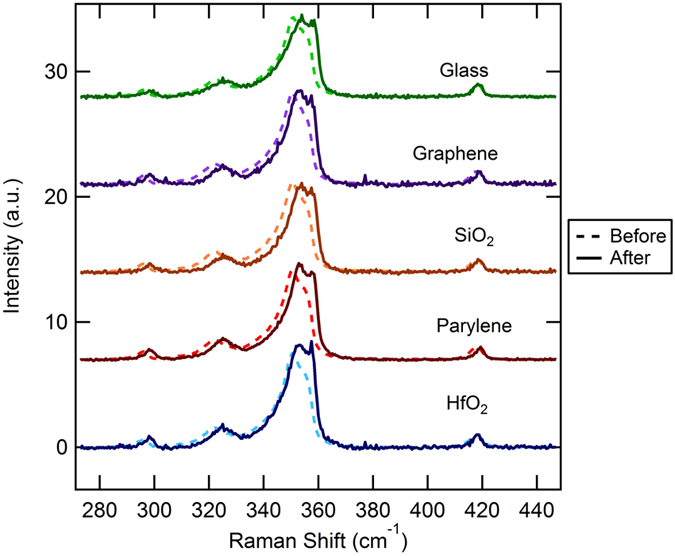
Raman spectra before and after transfer for each substrate type used. The spectra displayed are averaged amongst 7–11 flakes for each substrate type. For each transfer process the initial substrate was as grown on a SiO_2_ substrate.

Qualitative results from the transfer process are shown in Fig. 3 which shows the averaged Raman spectra from several flakes for each substrate before transfer (on as-grown substrates) and after transfer to a new substrate (after transfer to post annealing is shown in supplementary information Fig. S2.1). The spectra are background subtracted using the adaptive baseline correction algorithm (arPLS)^52^ and normalized to the out–of-plane A’_1_ peak. As can be observed for all samples, there is an obvious blue shift after transfer in the in-plane modes in the 290 to 360 cm^−1^ range and a slight blue shift in the out–of-plane A’_1_ peak. Qualitatively the peak heights in the 350–360 cm^−1^ range also show an increase with the E’(Γ) peak, at 354 cm^−1^, becoming more prominent after transfer and in one case, HfO_2_, the E’ peak appears to have become larger than the 2LA(M) peak.

In order to extract quantitative information from each substrate type, the spectrum for each flake transferred and measured is fit with Lorentzians in the range of 280 cm^−1^ to 440 cm^−1^, fitting specifically the 2LA(M)-2E”(Γ) (294 cm^−1^), 2LA(M)-E”(Γ) (320 cm^−1^), E’(M) (341 cm^−1^), 2LA(M) (350 cm^−1^), E’(Γ) (354 cm^−1^), and A’_1_ (418 cm^−1^) peaks, as by Su *et al*.^15^ and Gong *et al*.^42^. Results focus on analysis and comparison of the 2LA(M), E’(Γ), and A’_1_ peaks in terms of frequency, full width at half maximum (FWHM), and intensity ratio. The peak fitting results from each flake are averaged together for each substrate at each step, the actual fit values for each flake are provided in supplementary information Figs S3.1–S3.9. Additional plots for a sample which was not transferred, but was thermally annealed is shown in supplementary information Fig. S4.1. For all of the following plots of the averaged fit values, results before transfer are plotted in red, results after transfer in blue, and results post annealing in green.

**Figure 4:**
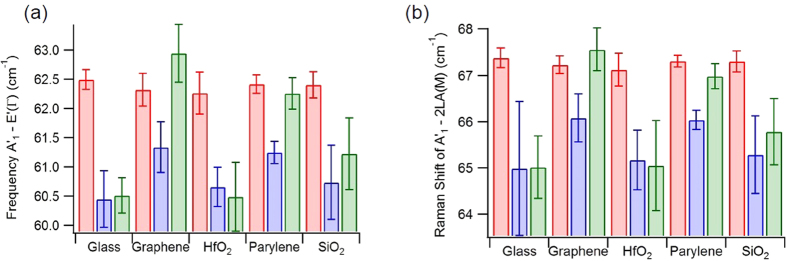
Bar graphs of average peak distance for WS_2_ flakes on different substrates, from before (red) to after transfer (blue) and after annealing (green). (**a**,**b**) Display the average distance between the 2LA(M) to A’_1_, and A’_1_ to E’(Γ), respectively. The error presented was calculated as the standard deviation of the mean of the flake fit values for each substrate type.

A widely used metric for determining layer thickness is to look at the difference between the locations of the A’_1_ and E’(Γ) peaks^4^,^34^,^40^,^41^, with the A’_1_ and 2LA(M) peaks showing similar behavior^34^,^44^. Figure 4(a,b) show that the flakes are monolayer before annealing based on the general reported value for the separation, approximately 62.5 cm^−1^ for A’_1_ to E’(Γ) and approximately 67 cm^−1^ for A’_1_ to 2LA(M)^34^. For all substrates after transfer both peak distances fall below previously reported values^34^. Post annealing, the separation stays decreased for glass, HfO_2_, and SiO_2_, while for graphene and parylene-C the values increase approximately back to their initial, before transfer, value. This further indicates that a careful and comprehensive method must be used to properly identify monolayer flakes using Raman spectroscopy as discussed in A. A. Mitioglu *et al*.^34^.

**Figure 5:**
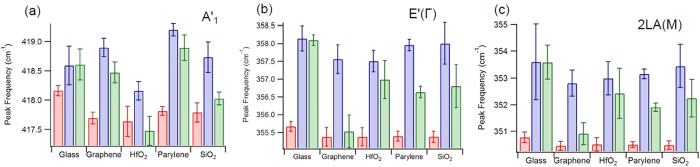
Bar graphs of the average peak location for WS_2_ flakes on different substrates, from before (red) to after transfer (blue) and after annealing (green), for the Raman peaks studied, (**a**) 2LA(M), (**b**) E’(Γ), and (**c**) A’_1_. The error presented was calculated as the standard deviation of the mean of the flake fit values for each substrate type.

The frequencies of the Raman peaks are shown in Fig. 5 for each substrate. For all peaks analyzed there is a blue shift after transfer. As reported in previous literature^15^,^18^, the overall blue shift after transfer is likely caused by a release of strain from the growth process. In comparing the peak locations to previously reported results^24^, we infer that the flakes were likely strained up to 1.5 percent as-grown and the strain was released after transfer. The post annealing shifts are, however, more likely the result of the flake interacting with the substrate. The annealing process, as shown in the AFM images in Fig. 2d–f and supported by the literature^9^,^21^,^51^, removes residual PMMA and water while promoting surface adhesion. Therefore, the post annealing results allow us to separate out the effects from the transfer process and analyze the substrate effects on the flakes.

For the out-of-plane A’_1_ peak position, which is most directly related to substrate interaction^13^,^14^,^21^,^41^,^51^, Fig. 5(a), the peak positions post annealing show a large variation between substrates. On SiO_2_, parylene-C, and graphene substrates there is a significant blue shift in the A’_1_ peak of approximately 1 cm^−1^ compared to as-grown flakes. The A’_1_ positions for HfO_2_ and SiO_2_ show blue shifts after flake transfer and then return to near their original value after annealing. These shifts caused by the substrates can come from several sources including: surface charge from leftover PMMA or the surface itself, re-straining of the flakes, surface adhesion, and defects. In order to rule out some of these effects we look at the in-plane modes, which are known to be less sensitive to substrate charge and surface adhesion and primarily affected by defects and strain^53^.

For the in-plane E’(Γ) and 2LA(M) peaks, Fig. 5(b,c) respectively, there is a large blue shift, on the order of 2 to 3 cm^−1^ after transfer. Post annealing, the blue shift remains the same for glass, decreases to approximately 1 to 2 cm^−1^ for HfO_2_, parylene-C, and SiO_2_, and goes back to near the pre-transfer value for graphene.

We can determine the likely dominant effects for these Raman peak shifts by comparing all three peaks collectively. For all substrates we rule out defects as dominant factor, as defects would introduce a red shift, not the blue shift which was observed after transfer^27^,^28^. The 2LA(M) to A’_1_ ratio, Fig. S6.1(b), also supports this conclusion as data show a small trend of increasing ratio for each substrate after transfer as opposed to a decrease^26^,^27^. For defects after annealing, we show in the supplement, Fig. S4.1, that the annealing process does not alter the peaks in as grown flakes.

In the case of the glass substrate, no new peak shifts are seen post annealing. This shows that immediately after transfer, the flakes have likely returned to an unstrained state and are unaffected by the substrate. That there is no substrate interaction, is evident from the A’_1_ peak which would be shifted by surface charge and adhesion. This result also suggests that any remaining PMMA from before or after annealing, likely has a minimal effect on the flakes.

For the graphene substrate, which shows a red shift for in-plane modes and a slight red shift in the A’_1_ peak, the flakes have likely been re-strained post annealing. This is supported by the slight increase in the FWHM of the E’(Γ), Fig. S5.1(b), which has been observed to soften and split under applied strain^24^. Similar results have been observed on WS_2_ on graphene^18^ using photo-luminescence (PL) measurements, where a strain related PL red shift was observed after the use of a specialized transfer method to increase surface coupling, similar to the red shift observed in our data post annealing.

HfO_2_ has a small red shift in the in-plane modes, but a comparably large red shift in the out-of-plane mode. A lack of in-plane shift combined with a shift in the out-of-plane peak indicates that surface charge is the dominant factor^53^. For parylene-C, the red shift after annealing is small, but primarily affects the in-plane modes, showing that, similar to graphene, this substrate largely re-strains the flakes. This is supported by a similar, but smaller increase in the FWHM of the E’(Γ) peak when compared to the graphene substrate. SiO_2_ shows similar shifts to that observed with parylene, with the exception of the post annealing shift of the A’_1_, which shifts to a position near its pre-transfer location. This likely means there is some surface charge interaction. In summary, from this discussion, our empirical study indicates that we observe the largest strain interactions in graphene and parylene-C, and the largest surface charge interactions if HfO_2_ and SiO_2_, while in glass we observe no interactions with the substrate.

## Discussion

In conclusion both a facile transfer process and the existence of a substrate effect has been shown in monolayer WS_2_ flakes. The transfer process combines elements from previously published methods with the new use of Kapton tape to reduce the post transfer residue and is shown to not introduce significant defects. The changes in the Raman spectra of the flakes before, after transfer, and post annealing show the existence of in-plane strain from the growth process, the release of this strain after transfer, and the conforming of the flakes to their new substrate after the annealing process. The data for each substrate show a varying final state indicating the WS_2_ flakes interact differently with each one. Graphene is the most interesting as re-straining and increases in the intensity and hardening of the in-plane mode are observed. The results highlight the importance of careful analysis of Raman data as well as the differences in substrate effects on monolayer samples, paving the way for more informed device fabrication.

## Methods

### WS_2_ synthesis

Monolayer and multilayer WS_2_ flakes were synthesized by CVD using a method developed in a previous report^54^. A 3.1 mM solution of Ammonia Metatungstate (Sigma Aldrich, 358975) and 0.02 g/mL sodium cholate is spin coated at 4000 rpm on a SiO_2_/Si substrate. This chip is placed in the center of a 1 inch Lindberg Blue furnace and accompanied with a Sulfur chip placed upstream at a distance of 17 cm from previous chip. The furnace is then ramped up to 800 °C at a ramp rate of 70 °C/min. Once at 800 °C H_2_ is introduced at 5 sccm for 5 min. The H_2_ is then stopped and the furnace is rapidly cooled to room temperature.

### Raman Measurement

Raman spectroscopy was performed in ambient conditions using a 532 nm laser excitation wavelength with a 1 *μ*m spot size and 55 *μ*W incident power. Spectra were obtained with an 1800 lines/mm grating attached to a NT-MDT spectrometer, resulting in a spectral resolution of 0.5 cm^−1^.

## Additional Information

**How to cite this article:** Mlack, J. T. *et al*. Transfer of monolayer TMD WS_2_ and Raman study of substrate effects. *Sci. Rep.* **7**, 43037; doi: 10.1038/srep43037 (2017).

**Publisher's note:** Springer Nature remains neutral with regard to jurisdictional claims in published maps and institutional affiliations.

## Supplementary Material

Supplementary Information
