# Supplementary material for: Transfer of monolayer TMD WS2 and Raman study of substrate effects
Source: Sci Rep. 2017 Feb 21;7:43037. doi: 10.1038/srep43037 (PMC5318859; doi:10.1038/srep43037)
Supplement: Supplementary Information [file srep43037-s1.pdf]

Supporting Information

Transfer of monolayer TMD WS_2 and Raman study of substrate effects

*Jerome T. Mlack[1‡], Paul Masih Das[1‡], Gopinath Danda[1,2‡], Yung-Chien Chou[3], Carl Naylor[1], Zhong Lin[4,5], Néstor Perea-López[4,5], Tianyi Zhang[6], Mauricio Terrones[4,5,6,7], A. T. Charlie Johnson[1], Marija Drndić[1]**

[1] Department of Physics and Astronomy, University of Pennsylvania, Philadelphia, Pennsylvania 19104, United States

[2] Department of Electrical and Systems Engineering, University of Pennsylvania, Philadelphia, Pennsylvania 19104, United States

[3] Department of Materials Science and Engineering, University of Pennsylvania, Philadelphia, Pennsylvania 19104, United States

[4] Department of Physics, The Pennsylvania State University, University Park, Pennsylvania 16802, United States

[5] Center for 2-Dimensional and Layered Materials, The Pennsylvania State University, University Park, Pennsylvania 16802, United States

[6]Department of Materials Science and Engineering, The Pennsylvania State University, University Park, Pennsylvania 16802, United States

[7]Department of Chemistry, The Pennsylvania State University, University Park, Pennsylvania 16802, United States

‡These authors have contributed equally.

*Corresponding author: Marija Drndić, drndic@physics.upenn.edu

Table of Contents:

1. **Additional images of transfer process**
2. **Averaged Raman data for After Transfer to After Annealing**
3. **Fit values for all Raman data for every substrate type**
4. **Raman data from annealing as-grown flakes**
5. **Full-width at half maximum comparisons**
6. **Intensity ratio comparisons**

Section 1: Additional images of transfer process

.

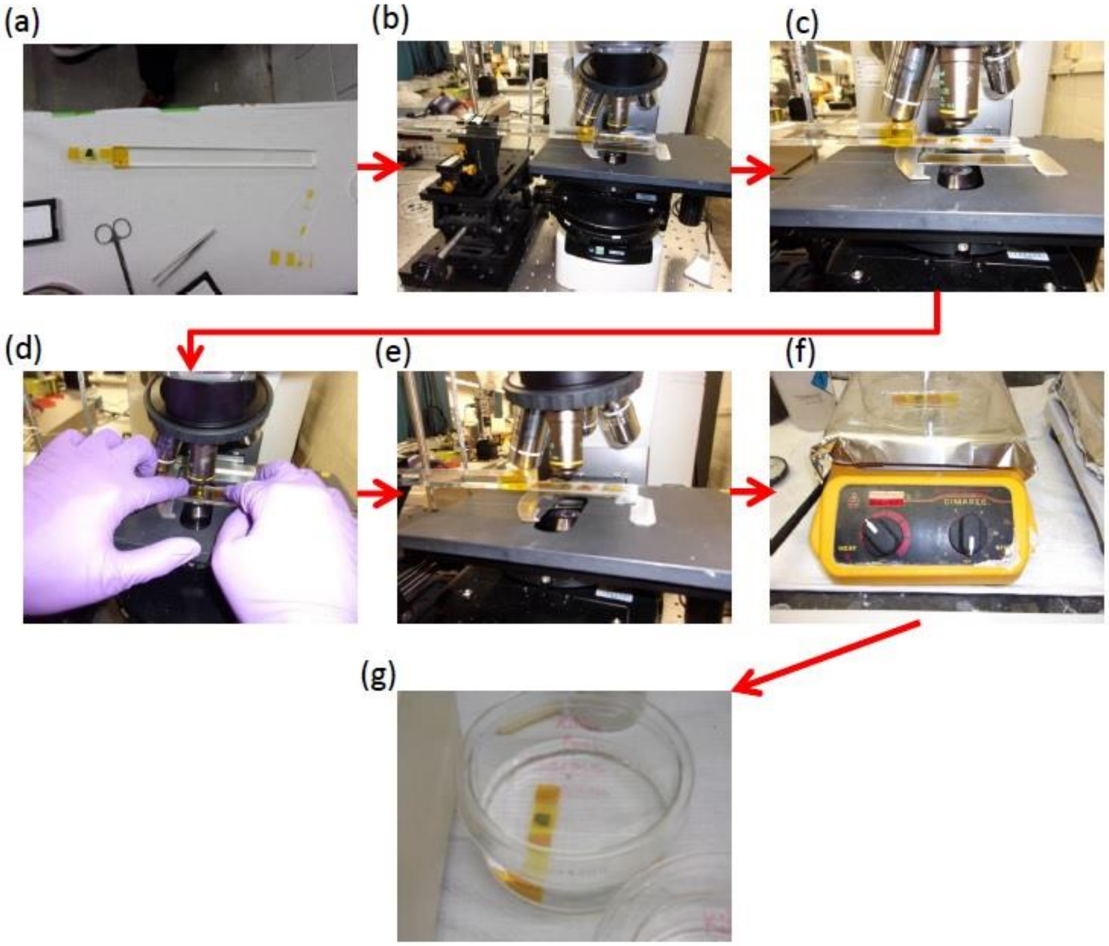

Figure SI 1.1: Pictures of the transfer process operating in an upside down process. (a) Final substrate, in this case silicon with 100 nm silicon nitride, and a transparent SiN window in its center, is attached to double sided Kapton tape on a glass slide is attached to the positioning stick. (b) Positioning stick is attached to movable stage with micromanipulator and under the microscope. (c) Micromanipulator is used to focus the SiN window to match the position of prepositioned flakes on PMMA. (d) The sample substrate has been lowered onto the flakes and pressure is being applied to ensure contact between the substrate and flakes. (e) The assembly of the final substrate and flakes on PMMA is lifted of the microscope, attached together by the two Kapton tape pillars. (f) The assembly is baked on a hot plate in a fume hood at 175 C. (e) The assemply is left in Acetone overnight for removal of the PMMA.

Section 2: Averaged Raman data for After Transfer to After Annealing

Raman maps

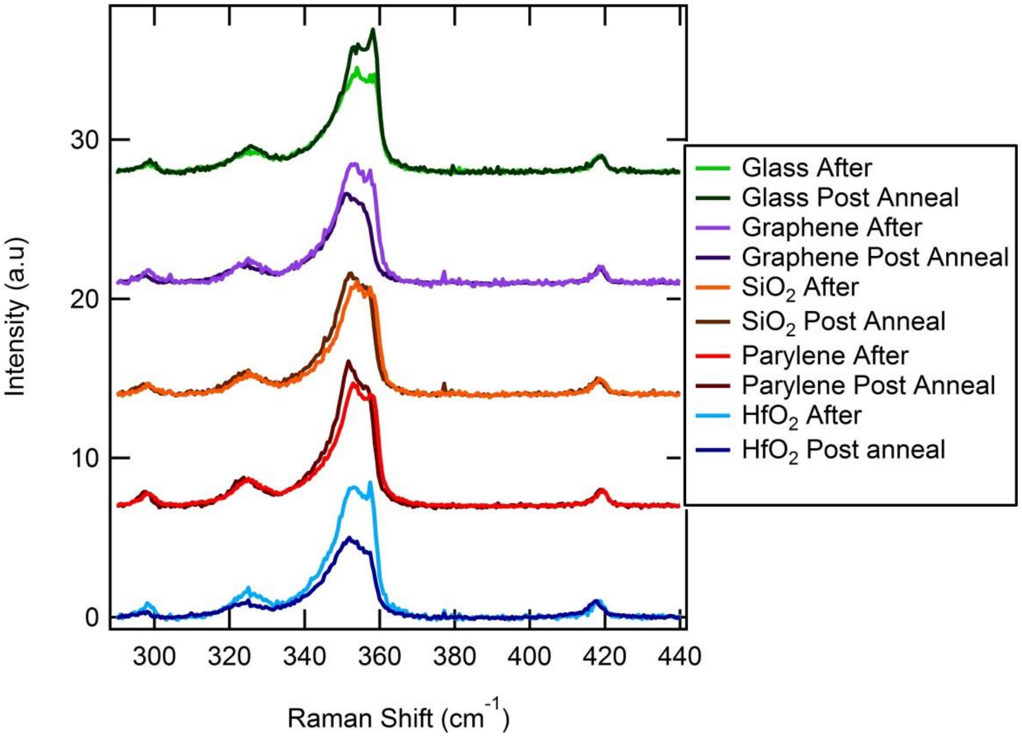

Figure SI 2.1. Raman spectra before and after transfer for each substrate type used. The spectra displayed are averaged amongst 7-11 flakes for each substrate type. For each transfer process the initial substrate was as grown on a SiO_2 substrate.

Section 3: Fit values for all Raman data for every substrate type

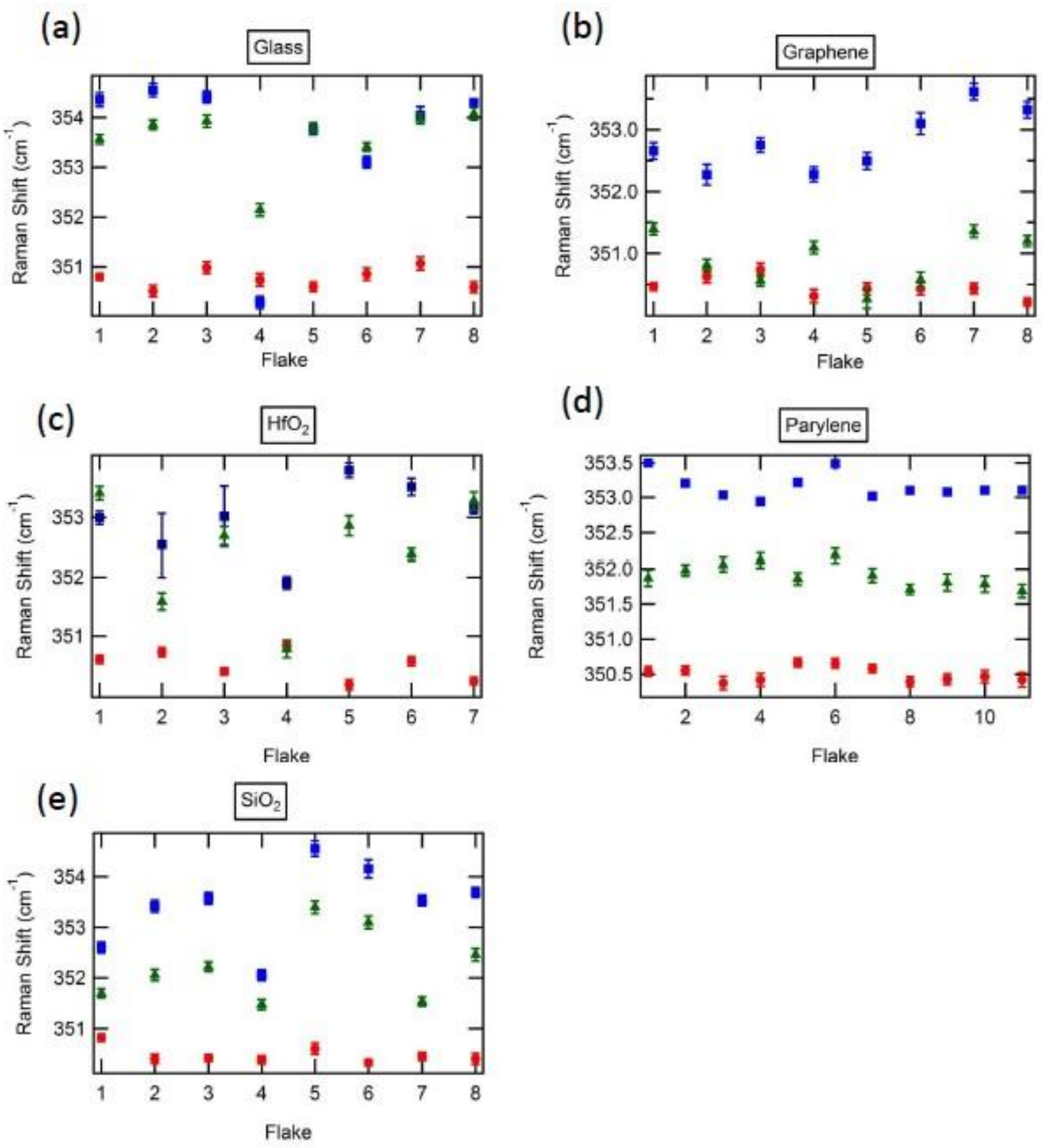

Figure SI 3.1. Raman shift fit values of the 2LA(M) peak for each individual flake measured on each substrate before(red circles), after transfer(blue squares), and post annealing(green triangles) . (a) Fit values for flakes transferred to Glass. (a) Fit values for flakes transferred to graphene. (a) Fit values for flakes transferred to HfO_2. (a) Fit values for flakes transferred to parylene-C. (a) Fit values for flakes transferred to SiO_2. Error bars are calculated during the fitting process.

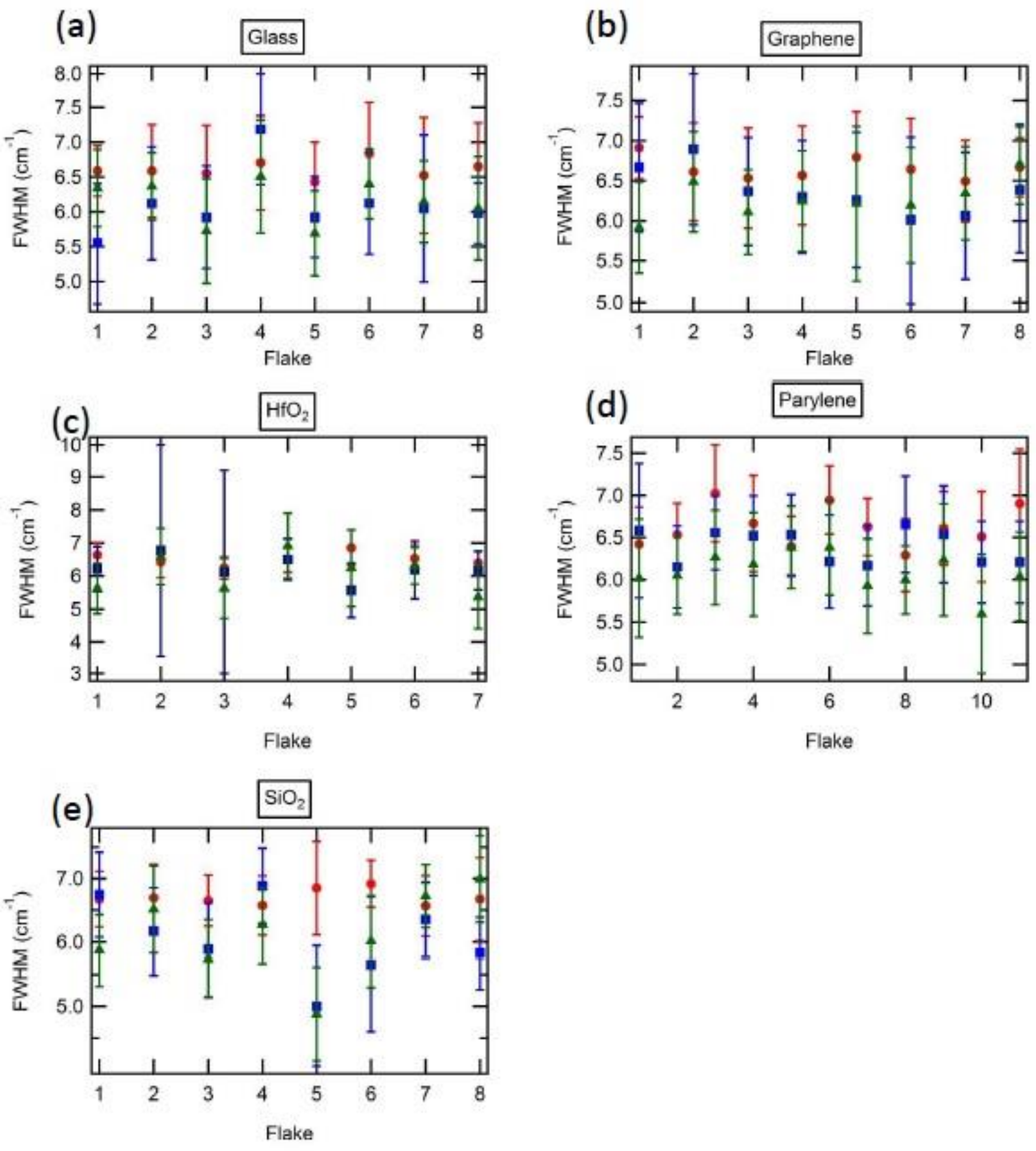

Figure SI 3.2. FWHM fit values of the 2LA(M) peak for each individual flake measured on each substrate before(red circles), after transfer(blue squares), and post annealing(green triangles) . (a) Fit values for flakes transferred to Glass. (a) Fit values for flakes transferred to graphene. (a) Fit values for flakes transferred to HfO_2. (a) Fit values for flakes transferred to parylene-C. (a) Fit values for flakes transferred to SiO_2. Error bars are calculated during the fitting process.

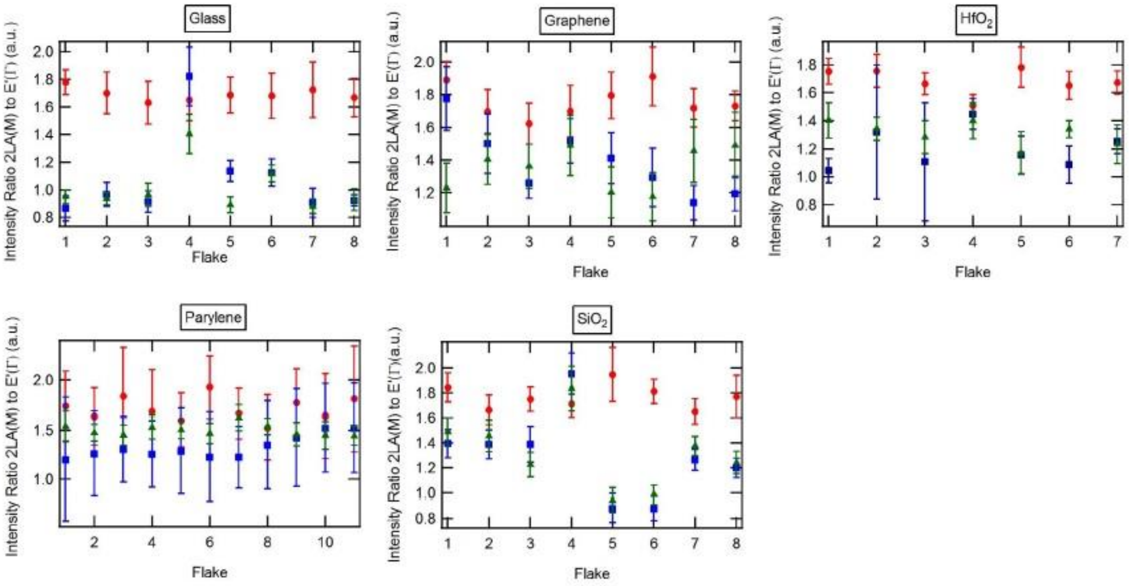

Figure SI 3.3. Intensity ratio of the fit values of the ratio of the 2LA(M) peak to the E`(Γ) peak for each individual flake measured on each substrate before(red circles), after transfer(blue squares), and post annealing(green triangles) . (a) Fit values for flakes transferred to Glass. (a) Fit values for flakes transferred to graphene. (a) Fit values for flakes transferred to HfO_2. (a) Fit values for flakes transferred to parylene-C. (a) Fit values for flakes transferred to SiO_2. Error bars are calculated from the errors for each peak height from the fitting process.

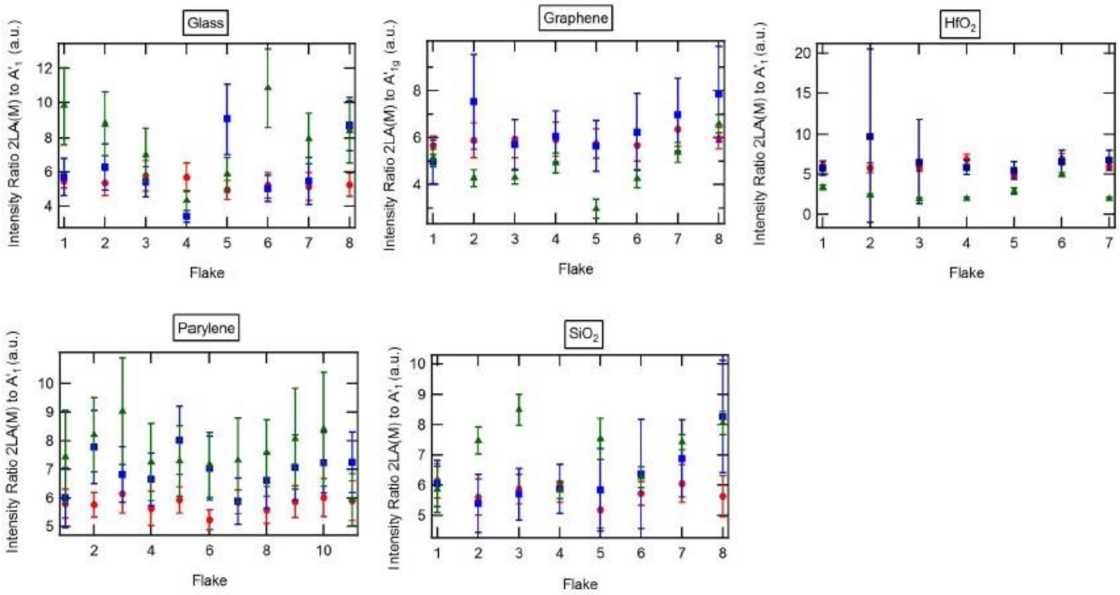

Figure SI 3.4. Intensity ratio of the fit values of the ratio of the 2LA(M) peak to the $A`_1(\Gamma)$ peak for each individual flake measured on each substrate before(red circles), after transfer(blue squares), and post annealing(green triangles) . (a) Fit values for flakes transferred to Glass. (a) Fit values for flakes transferred to graphene. (a) Fit values for flakes transferred to HfO_2. (a) Fit values for flakes transferred to parylene-C. (a) Fit values for flakes transferred to SiO_2. Error bars are calculated from the errors for each peak height from the fitting process.

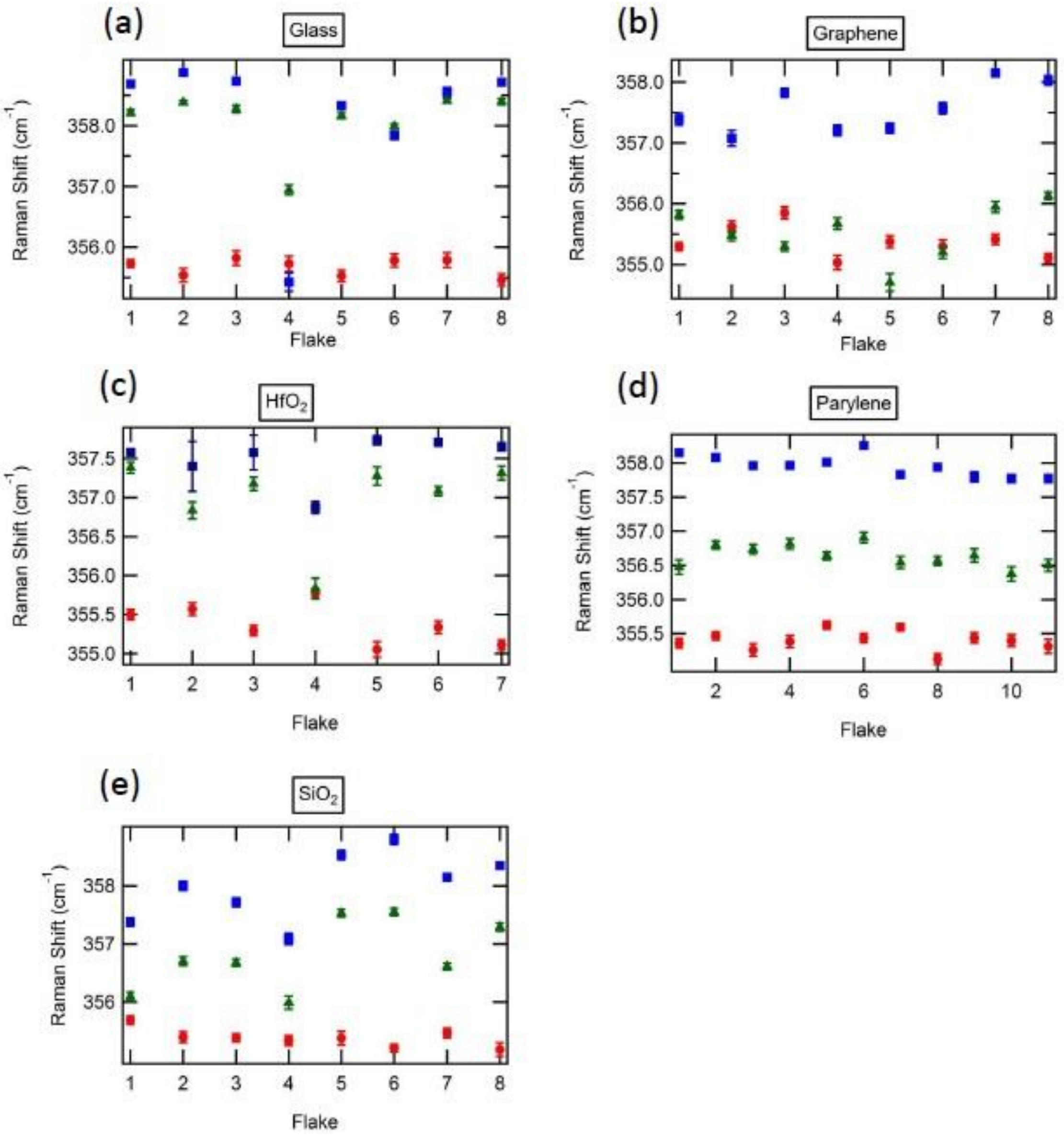

Figure SI 3.5. Raman shift fit values of the E`(Γ) peak for each individual flake measured on each substrate before(red circles), after transfer(blue squares), and post annealing(green triangles) . (a) Fit values for flakes transferred to Glass. (a) Fit values for flakes transferred to graphene. (a) Fit values for flakes transferred to HfO_2. (a) Fit values for flakes transferred to parylene-C. (a) Fit values for flakes transferred to SiO_2. Error bars are calculated during the fitting process.

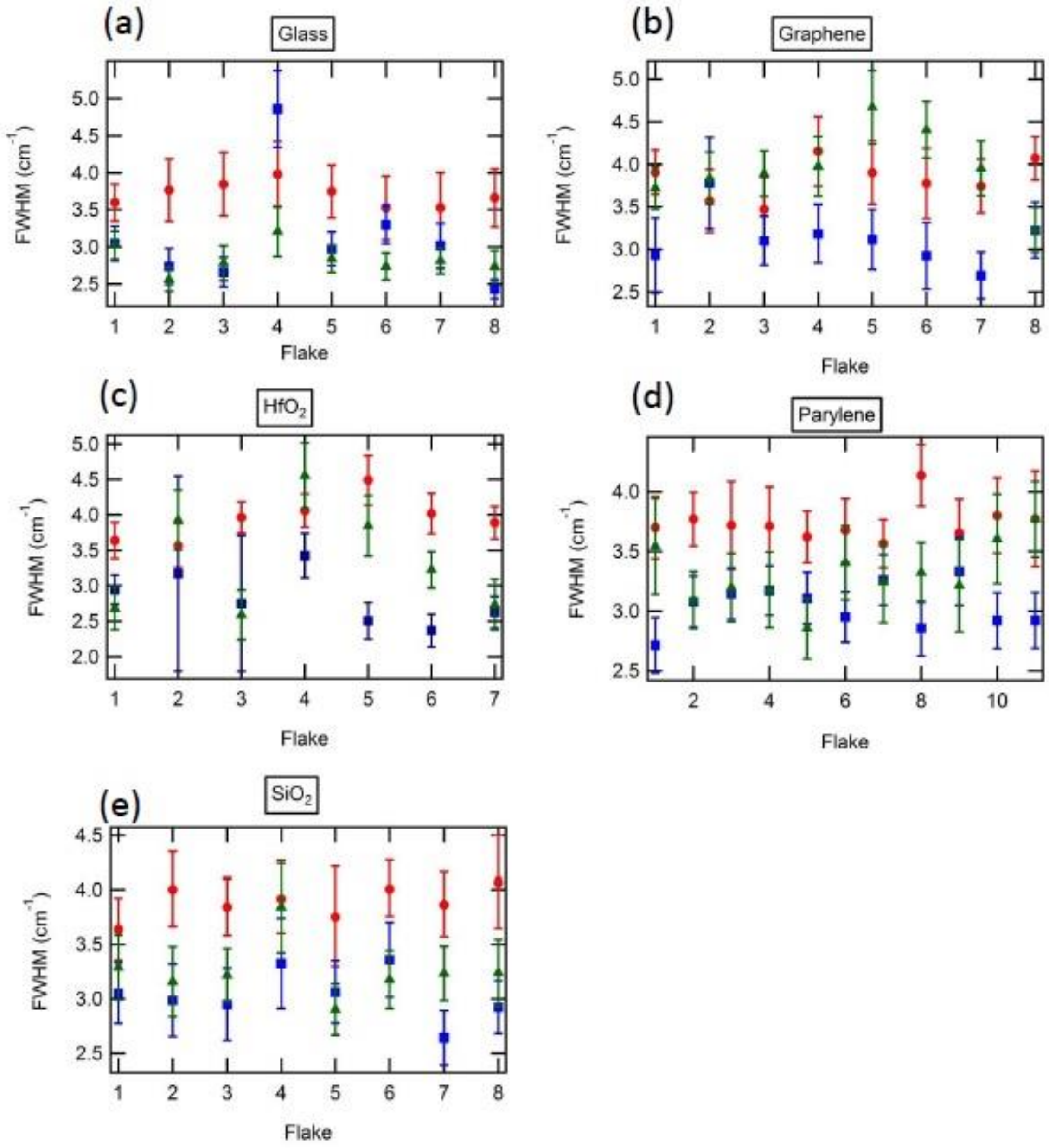

Figure SI 3.6. FWHM fit values of the E`(Γ) peak for each individual flake measured on each substrate before(red circles), after transfer(blue squares), and post annealing(green triangles) . (a) Fit values for flakes transferred to Glass. (a) Fit values for flakes transferred to graphene. (a) Fit values for flakes transferred to HfO_2. (a) Fit values for flakes transferred to parylene-C. (a) Fit values for flakes transferred to SiO_2. Error bars are calculated during the fitting process.

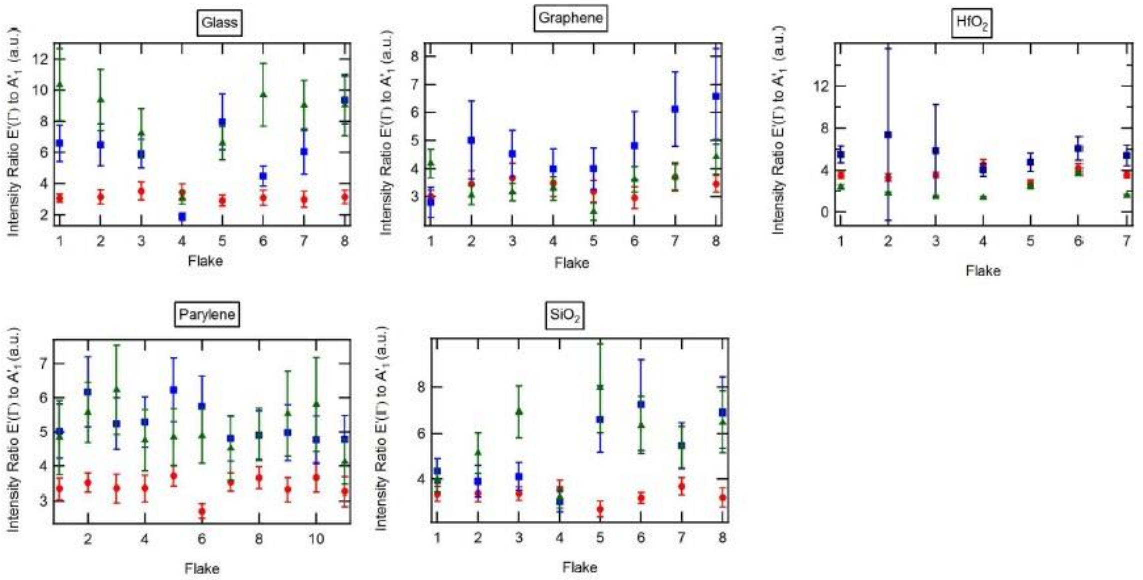

Figure SI 3.7. Intensity ratio of the fit values of the ratio of the E`(Γ) peak to the A`$_1$(Γ) peak for each individual flake measured on each substrate before(red circles), after transfer(blue squares), and post annealing(green triangles) . (a) Fit values for flakes transferred to Glass. (a) Fit values for flakes transferred to graphene. (a) Fit values for flakes transferred to HfO_2. (a) Fit values for flakes transferred to parylene-C. (a) Fit values for flakes transferred to SiO_2. Error bars are calculated from the errors for each peak height from the fitting process.

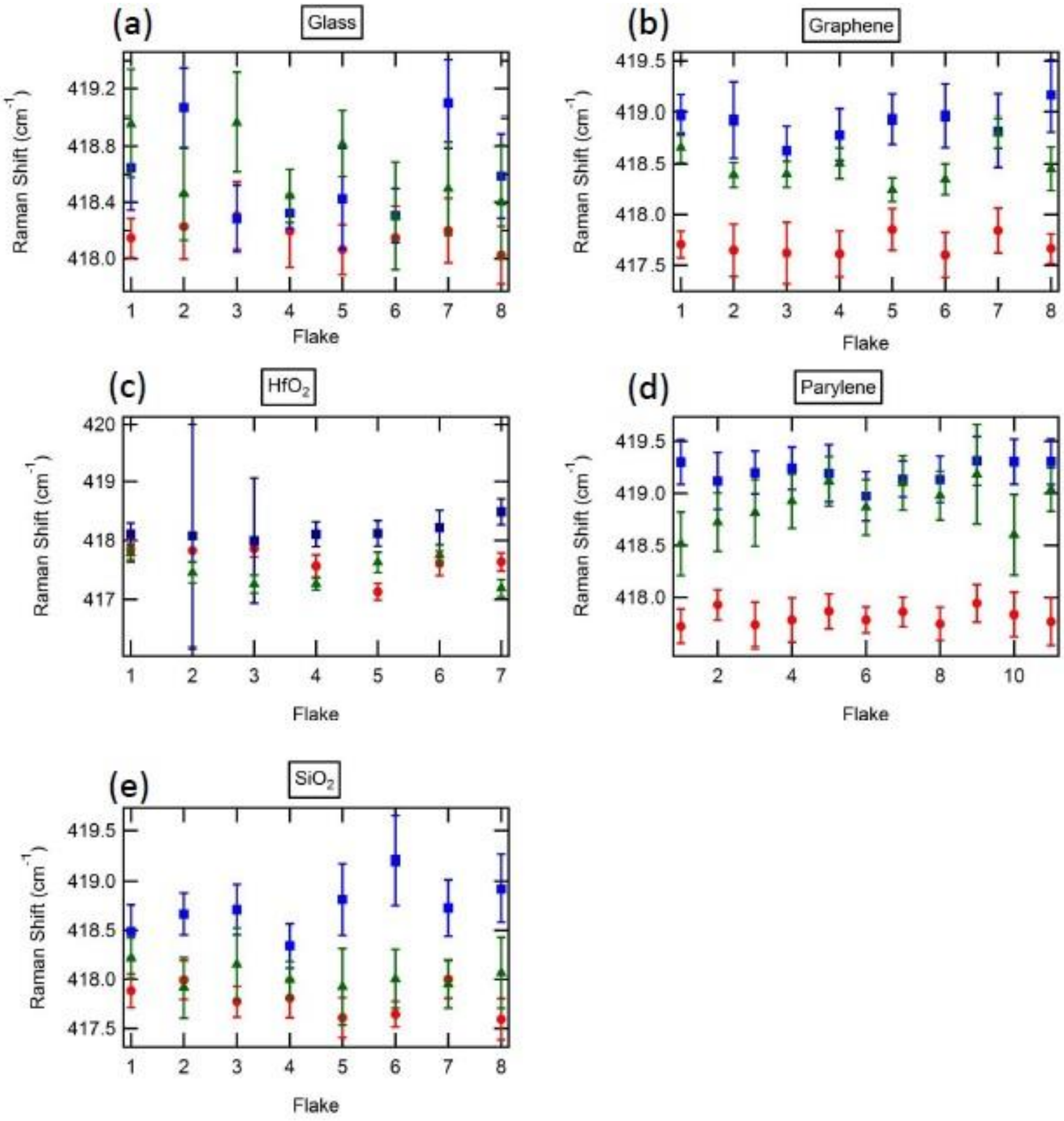

Figure SI 3.8. Raman shift fit values of the $A`_1(\Gamma)$ peak for each individual flake measured on each substrate before(red circles), after transfer(blue squares), and post annealing(green triangles) . (a) Fit values for flakes transferred to Glass. (a) Fit values for flakes transferred to graphene. (a) Fit values for flakes transferred to HfO_2. (a) Fit values for flakes transferred to parylene-C. (a) Fit values for flakes transferred to SiO_2. Error bars are calculated during the fitting process.

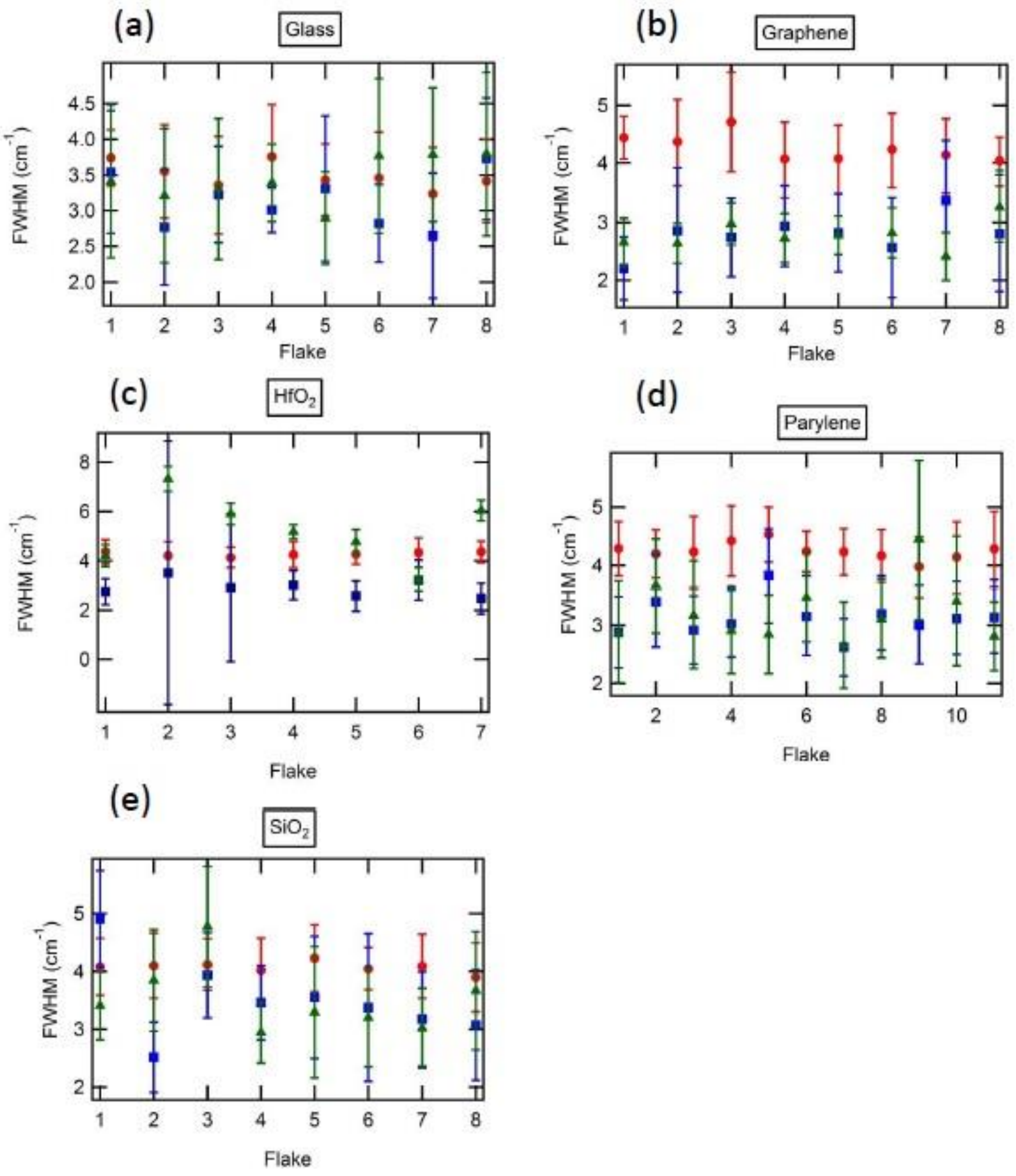

Figure SI 3.9. FWHM fit values of the $A`_{1}(\Gamma)$ peak for each individual flake measured on each substrate before(red circles), after transfer(blue squares), and post annealing(green triangles) . (a) Fit values for flakes transferred to Glass. (a) Fit values for flakes transferred to graphene. (a) Fit values for flakes transferred to HfO_2. (a) Fit values for flakes transferred to parylene-C. (a) Fit values for flakes transferred to SiO_2. Error bars are calculated during the fitting process.

Section 4: Raman data from annealing as-grown flakes

In order to check that the annealing process is not itself causing changes in the flakes, the Raman spectra of a sample of as-grown flakes was measured before and after an annealing step with no transferring involved. As can be observed for all plots in Fig. SI 4.1 there is no change in the properties from before(red circles) to after annealing(green triangle). This indicated that the annealing process does not induce and major defect in the flakes and that the changes observed in the plots in the main text and Figures SI 2.1 and SI 3.1-3.9 are likely from changes in the material structure, not material quality.

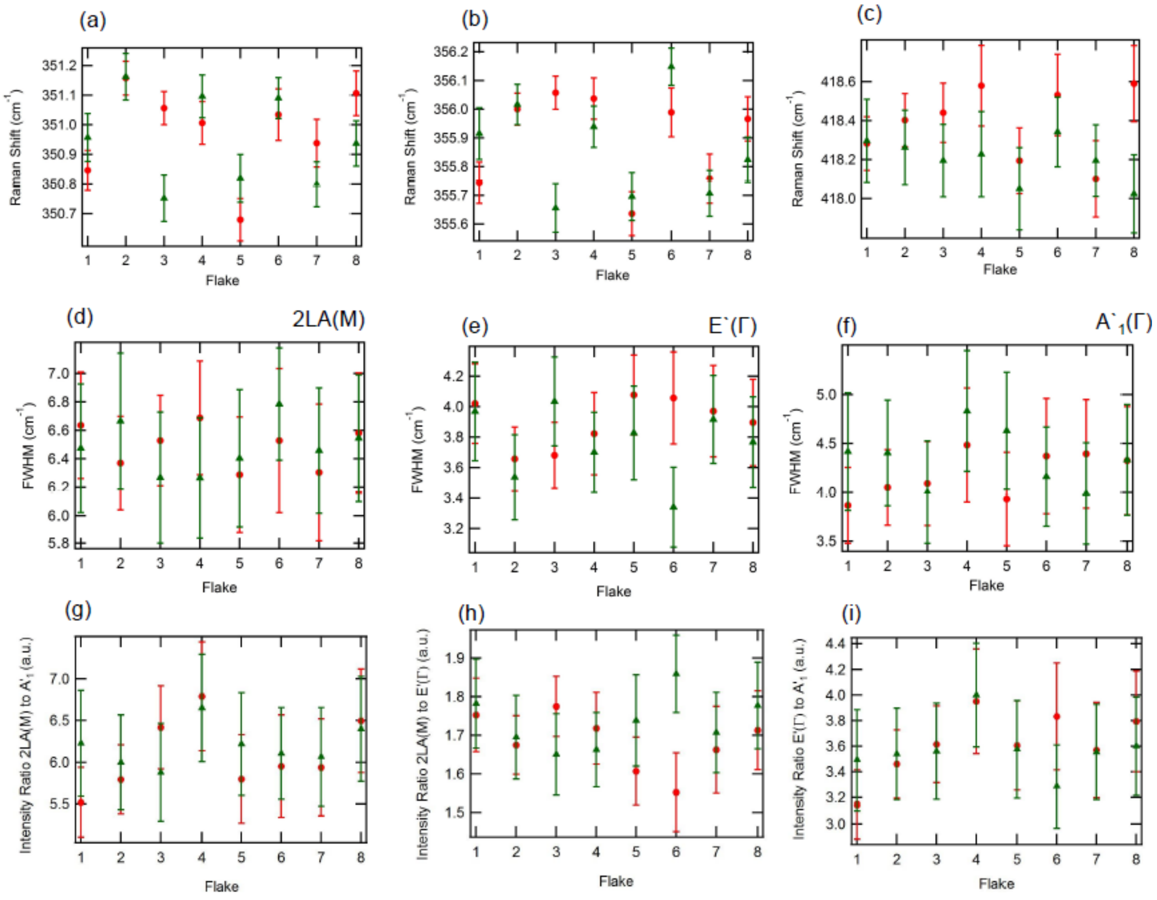

Figure SI 4.1. Raman shift, FWHM, and intensity ratios for a sample of as-grown flakes before(red circles) and after annealing(green triangles) with no transferring. (a) Raman shift

of 2LA(M) peak. (b) Raman shift of E`(Γ) peak. (c) Raman shift of A`$_1$(Γ) peak. (d) FWHM of 2LA(M) peak. (e) FWHM of E`(Γ) peak. (f) FWHM of A`$_1$(Γ) peak. (g) Intensity ratio of 2LA(M) to A`$_1$(Γ) peak. (h) Intensity ratio of 2LA(M) to E` peak. (i) Intensity ratio of E`(Γ) to A`$_1$(Γ) peak.

Section 5: Full-width at half maximum comparisons

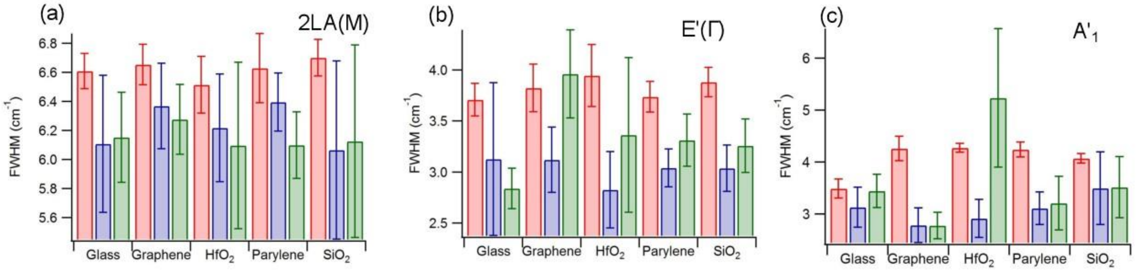

Figure SI 5.1. Bar graphs of full width at half maximum for WS_2 flakes on different substrates, from before(red) to after transfer(blue) and after annealing(green), for each Raman peak studied. (a) 2LA(M), (b) E'(Γ) and (c) A'$_1$. The error presented was calculated as the standard deviation of the mean of the flake fit values for each substrate type.

Section 6: Intensity ratio comparisons

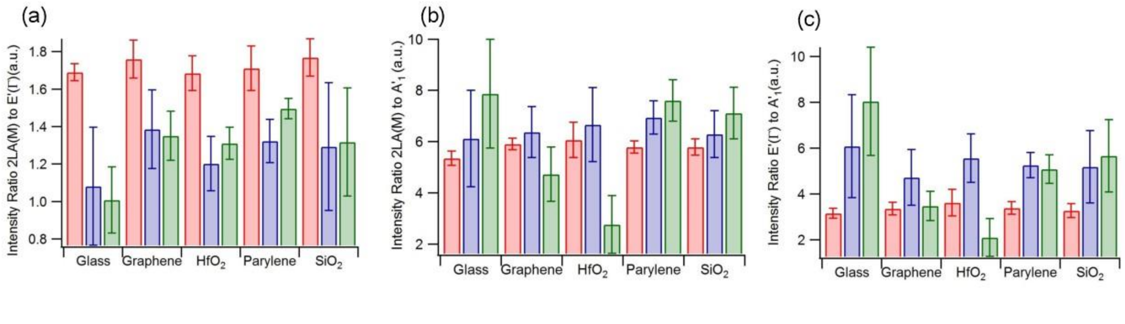

Figure SI 6.1. Average intensity ratio between different peaks for WS_2 flakes on different substrates, from before(red) to after transfer(blue) and after annealing(green). (a) 2LA(M),

(b) $E'(\Gamma)$ and (c) A'_1. The error presented was calculated as the standard deviation of the mean of the flake fit values for each substrate type.
